# The complete mitochondrial genome of *Micromus paganus* (Linnaeus, 1767) (Neuroptera: Hemerobiidae: Microminae) with phylogenetic analysis

**DOI:** 10.1080/23802359.2021.1934169

**Published:** 2021-06-03

**Authors:** Liming Wang, Cong Li, Ruyue Zhang, Pan Yi, Yuyu Wang, Xingyue Liu

**Affiliations:** a College of Plant Protection, Hebei Agricultural University, Baoding, China; b Department of Entomology, China Agricultural University, Beijing, China

**Keywords:** *Micromus paganus*, mitochondrial genome, phylogeny

## Abstract

The complete mitochondrial (mt) genome of *Micromus paganus* (Linnaeus, 1767) (Neuroptera: Hemerobiidae: Microminae) was assembled and the phylogenetic analysis of Chrysopoidea was conducted. The mt genome was 16,607 bp long including 13 protein-coding genes (PCGs), 22 tRNA genes, 2 rRNA genes, and a control region (CR). Twelve PCGs started with typical ATN, but *COI* initiated with TCG. The control region was 1335 bp long and the base composition was 89.66% of A + T. Phylogenetic analysis revealed that *M. paganus* was the sister group to *Micromus* sp. *+ M. angulatus*. Hemerobiinae and Microminae were recovered monophyletic with high support values. However, the monophyly of Drepanepteryginae was not recovered, which needed more samplings from this subfamily in the further study. The closer relationship between Microminae and Drepanepteryginae was supported. Hemerobiidae was demonstrated monophyletic and being the sister group to Chrysopidae.

*Micromus paganus* (Linnaeus, 1767) belongs to Microminae of Hemerobiidae, the third largest family of Neuroptera, which can prey on various pests (such as aphids, mites, scale insects and whiteflies) with great potentials in biological control (Linnaeus 1767; Yang 1981; Aspöck et al. 2001; Yang et al. 2018). There are about 650 known species of eleven subfamilies in the world (Oswald 2019). The mitochondrial (mt) genome is an important marker for the analysis of molecular evolution and has been widely used in the study of the phylogenetic relationships of Insecta at different levels (Mao et al. 2015).

In this study, we sequenced and annotated the complete mt genome of *M. paganus* (GenBank Accession no. MW800748). The specimen was collected by Xingyue Liu on 2018-9-5 at Wuling Mountain, Hebei Province, China (E117°29'12“, N40°33'50“). Total genomic DNA was extracted from thoracic muscle using the DNeasy Blood & Tissue kit (Qiagen, Hilden, Germany). The voucher specimen and DNA was kept in Hebei Agricultural University Museum (contact Dr Yuyu Wang, E-mail: hebau_entmus@126.com) under the voucher number HEM001. It was sequenced by Illumina NovaSeq 6000 platform with 150 bp paired-end reading strategy. Raw reads about 3 Gb were checked by FastQC 0.11.9 (Andrews 2020) and low-quality reads were filtered by Trimmomatic 0.32 (Bolger et al. 2014). The mt genome was assembled using IDBA-UD 1.1.3. (Peng et al. 2012) and annotated by MITOS Web Server (Bernt et al. 2013) and then checked by manual proofreading.

This mt genome was a traditional double-strain circular molecule with 16,607 bp long including 22 tRNA genes, 13 protein-coding genes (PCGs), 2 rRNA genes, and a control region (CR). Meanwhile, 23 genes were encoded at the major strand while the remaining 14 genes were encoded at the minor strand. The overall base composition was 39.77% for A, 12.60% for C, 8.88% for G, and 38.74% for T. Twelve PCGs used the typical initiation codon ATN, while *COI* used TCG as the start codon. There were six genes ended with the incomplete stop codon (T-tRNA for *COI*, *ND2*, *ND5*, *CytB* and TA-tRNA for *COIII*, *ND4*) and seven PCGs terminated with the stop codon TAA. The length of tRNAs varied from 64 bp to 72 bp. The length of *rrnL* and *rrnS* was 1327 bp and 790 bp, respectively. The CR was 1335 bp in length with an A + T content of 89.66%.

The phylogenetic trees were reconstructed by MrBayes 3.2.2 (Ronquist et al. 2012) and RAxML 8.2.4 (Stamatakis 2014) based on the first and second codon positions of the PCGs inferring the same topology (Figure 1). *Ditaxis biseriata* (NC_013257) and *Euclimacia badia* (NC_039773) from Mantispidae were selected as outgroups. Phylogenetic analysis showed that *M. paganus* grouped with *Micromus* sp. + *M. angulatus*, all belonging to *Micromus* genus with high support values (PP = 1, BS = 100). Hemerobiinae and Microminae were recovered as monophyletic. Drepanepteryginae was recovered being the sister group to Microminae. However, the monophyly of Drepanepteryginae was not recovered, which needed further studies with more comprehensive samplings. Hemerobiidae was demonstrated monophyletic being the sister group to Chrysopidae.

**Figure 1.**
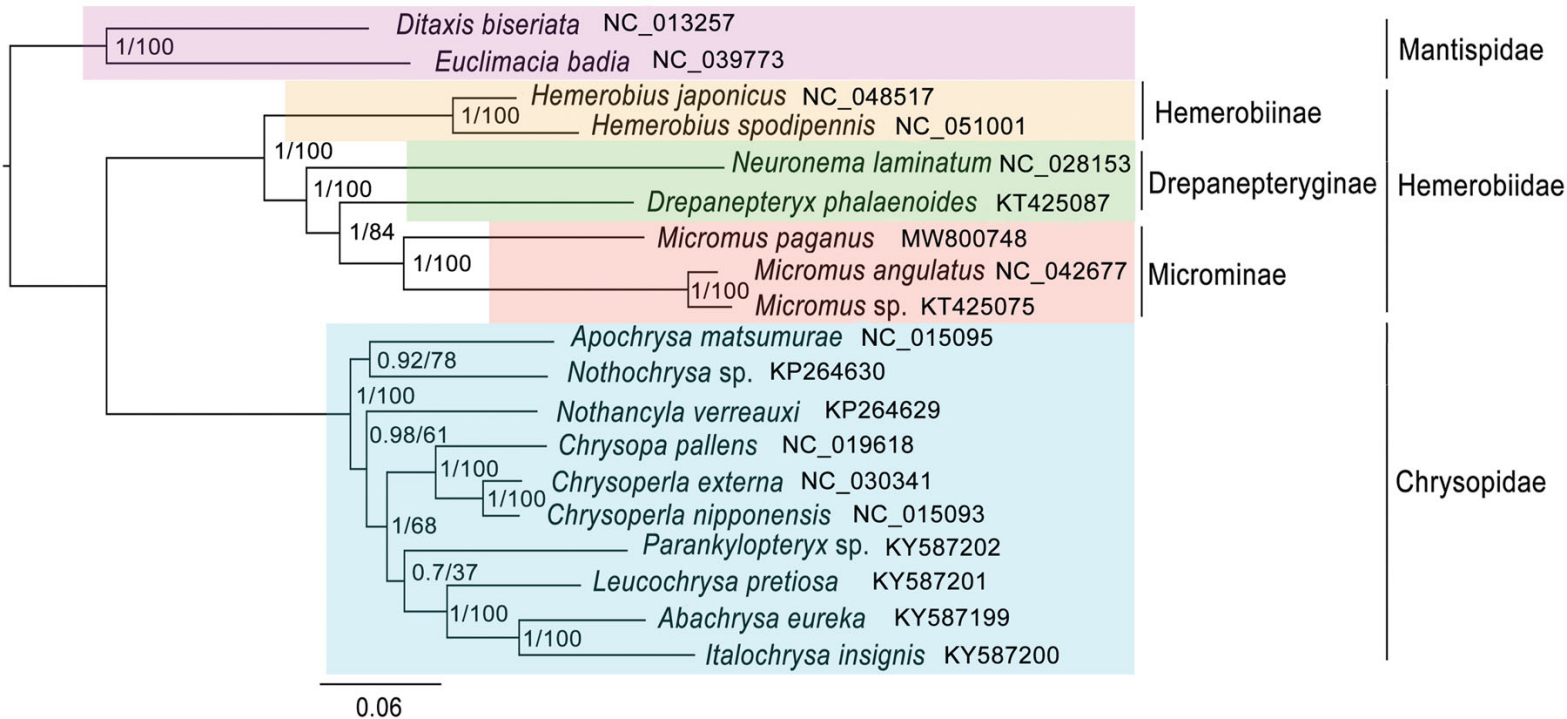
Phylogenetic relationships based on the first and second codon positions of 13 protein-coding genes inferred from RAxML and MrBayes. The nodal numbers indicates the posterior probability (left) and the bootstrap support values (right). Genbank accession numbers for the sequences are listed next to the species names.

## Data Availability

The genome sequence data that support the findings of this study are openly available in GenBank of NCBI at https://www.ncbi.nlm.nih.gov under the accession MW800748. The associated BioProject, SRA, and Bio-Sample numbers are PRJNA707120, SRR14066566, and SAMN18437193, respectively.
